# Endothelial Glycocalyx as a Regulator of Fibrotic Processes

**DOI:** 10.3390/ijms22062996

**Published:** 2021-03-15

**Authors:** Valentina Masola, Gianluigi Zaza, Arduino Arduini, Maurizio Onisto, Giovanni Gambaro

**Affiliations:** 1 Department of Biomedical Sciences, University of Padova, Viale G. Colombo 3, 35121 Padova, Italy; valentina.masola@unipd.it; 2 Division of Nephrology and Dialysis, Department of Medicine, Piazzale A. Stefani 1, 37126 Verona, Italy; gianluigi.zaza@univr.it; 3 R&D Department, CoreQuest Sagl, Via Cantonale 18, 6928 Manno, Switzerland; a.arduini@corequest.ch

**Keywords:** endothelium, glycocalyx, fibrosis

## Abstract

The endothelial glycocalyx, the gel layer covering the endothelium, is composed of glycosaminoglycans, proteoglycans, and adsorbed plasma proteins. This structure modulates vessels’ mechanotransduction, vascular permeability, and leukocyte adhesion. Thus, it regulates several physiological and pathological events. In the present review, we described the mechanisms that disturb glycocalyx stability such as reactive oxygen species, matrix metalloproteinases, and heparanase. We then focused our attention on the role of glycocalyx degradation in the induction of profibrotic events and on the possible pharmacological strategies to preserve this delicate structure.

## 1. Introduction

The endothelium was once only known as the cellular internal monolayer of blood and lymphatic vessels, but now it is also recognized as a dynamic organ. First of all, endothelial cell phenotype is different in different organs and districts, and this reflects specific functions [1]. For instance, in the kidney, endothelial cells of the large vessels are a continuous layer kept together by intercellular junctions, whereas glomerular endothelial cells and endothelial cells of peritubular capillaries are highly fenestrated [2]. Endothelial cells are covered by a gelatinous layer called the “glycocalyx”, which represents an important element of the vascular barrier [3].

In the last few years, it has appeared that (1) a great deal of endothelial functions are modulated and mediated by the glycocalyx, (2) the integral endothelial surface layer is an important element in tissue homeostasis, and (3) alterations of this structure are involved in several pathophysiological conditions: sepsis as well as chronic cardiovascular, renal, and metabolic diseases [4,5,6,7].

In this review, we will describe the composition and function of endothelial glycocalyx together with the mechanisms responsible for its degradation. We will also focus on the recent findings as to the role of the endothelial glycocalyx in the modulation of renal fibrosis. Finally, we will discuss the present and possible future strategies aimed at preserving this delicate structure.

## 2. Endothelial Glycocalyx Structure and Functions

The glycocalyx is synthesized by vascular endothelial cells and expressed on the endothelial cell surface [8], and it is mainly composed of membrane-binding proteoglycans (mainly syndecans 1, 2, and 4 and glypican 1), glycosaminoglycan (GAG) side-chains conjugated with the core protein of the proteoglycans, hyaluronan, glycoproteins, and adsorbed plasma proteins (such as albumin and antithrombin) [9].

Proteoglycans are a group of molecules composed of a core protein to which several GAGs are attached. GAGs are negatively charged unbranched polysaccharide chains made up of the repetition of disaccharide units. Depending on their core disaccharide units, GAGs are classified in heparin/heparan sulfate, chondroitin sulfate, dermatan sulfate, and keratan sulfate [10]. Since GAGs have sulfated groups, they are negatively charged, and this gives them the capacity to bind several growth factors and cytokines, thus protecting them from degradation [11]. Moreover, they create a gradient necessary for fluid transit and renal blood filtration [12,13]. Hyaluronan (a nonsulfated GAG) is found mainly in the luminal part of the glycocalyx, and it is not attached to a core protein but binds to surface receptors (e.g., CD44) [14].

Syndecans are central elements in endothelial cell homeostasis. Their interaction with ligands modulates endothelial cell growth and behavior [15]. Glypicans are important regulators of angiogenesis and coagulation [16,17]. The three groups of cell glycoproteins present in the endothelial glycocalyx are the selectin family, the integrin family, and the immunoglobulin superfamily [8]. E-selectin and P-selectin are both expressed by endothelial cells with different mechanisms and both modulate leukocyte–endothelial cell interactions [18,19,20]. Integrins are transmembrane receptors [21] that mediate platelet–endothelial cell interactions [22] and facilitate cell–extracellular matrix (ECM) adhesion [23]. The principal elements of immunoglobulin glycoproteins are intercellular adhesion molecules 1 and 2 (ICAM-1 and -2), vascular cell adhesion molecule 1 (VCAM-1), and platelet/endothelial cell adhesion molecule 1 (PECAM-1), and they regulate the binding of leukocytes and platelet endothelium [24].

The thickness of the glycocalyx was reported to be 4–500 nm, which depends on the measurement technique, and it was observed that there is a dense inner matrix layer associated with membrane-attached glycoproteins and a less-dense outer layer composed mainly of GAGs and plasma proteins [25]. Thickness is also modulated by the balance between synthesis caused by endothelial cells, GAG depolymerization, and proteoglycan shedding. Globally, the endothelial glycocalyx is a structure in dynamic equilibrium with the components of the flowing blood [26,27,28]. Interestingly, it has been proved that cerebral endothelial glycocalyx thickness is not homogeneous among pial arteries, penetrating arteries, and capillaries. Glycocalyx thickness was not correlated with the vessel diameter, but it might reflect the functional heterogeneity of the vessel type [29].

The main functions of the glycocalyx are the control of vessel tone in response to shear stress, the regulation of the permeability, coagulation and complement system, as well as the regulation of endothelial–blood cell interaction.

Proteoglycans such as syndecans and glypicans, but also hyaluronic acid, are mechanotransducers of the shear forces to endothelial cells [30]. For instance, when the flow becomes multidirectional, there is a modification of plasma proteins, cations, and cationic amino acids associated with GAGs and this could aid in syndecan-1 oligomerization and transduction of the signal via its intracellular domain, which is associated with linker molecules that connect them to the cytoskeleton [31]. The principal effects of shear stress are the alignment of endothelial cells in the shear direction (via mechanotransduction) and the production of nitric oxide (NO): a mediator that induces smooth muscle cell relaxation and, consequently, vasodilatation, and the reduction of shear stress [32].

The negatively charged endothelial glycocalyx represents an electrostatic barrier for plasma cells and proteins, like albumin. It also regulates permeability to water and small molecules and oxygen [12,33] and allows for the selective buffering of sodium ions [34]. It has been proved that enzymatic elimination of the majority of the endothelial glycocalyx modulates hydrostatic and oncotic pressure gradients between the lumen of the blood vessel and the interstitial space [35,36,37].

A functional glycocalyx represents an antithrombotic and anticoagulant surface as antithrombin III bound to GAGs modulate several coagulation factors [38] and GAGs interact with multiple complement elements of both: classic alternative and lectin pathways [39]. During inflammation, several cytokines activate the expression of tissue factors on endothelial cells. Tissue factor binds to and activates clotting factor VII, which via factor X, results in the generation of thrombin and conversion from fibrinogen to fibrin [40]. Thrombin can activate PAR1, which, in turn, induces the production of several cytokines and growth factors and causes platelet activation and aggregation. On endothelial cells, PAR1 activation rises endothelial permeability, and the von Willebrand factor (vWF) secretion from that participates in platelet recruitment. Platelets can also be activated by proinflammatory mediators and thus sustain fibrin formation [41,42].

**Figure 1:**
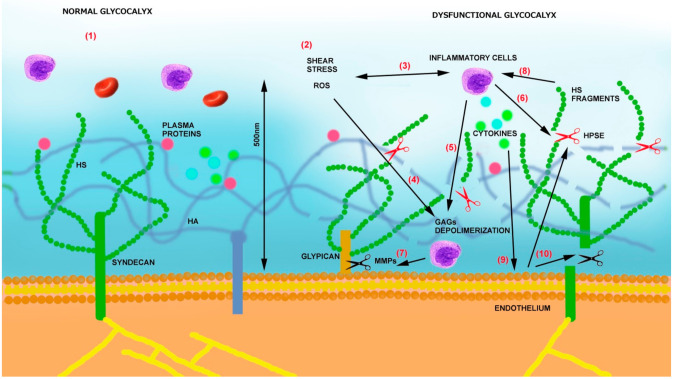
(1) Under physiological conditions, the glycocalyx represents a protective layer of proteoglycans (syndecans and glypicans), glycosaminoglycans (GAGs) (HS—heparan sulfate, HA—hyaluronic acid), and adsorbed plasma proteins. (2) In pathological situations such as atherosclerosis, ischemia, and diabetes, a series of stimuli impact glycocalyx integrity. (3) ROS and shear stress recruit activated inflammatory cells, and inflammatory cells release additional ROS. (4) ROS participates in GAG depolymerization, which aids in (5) leukocyte activation and macrophage extravasation. Activated inflammatory cells release (6) HPSE (red scissors) and (7) matrix metalloproteinases (MMPs) (black scissors). MMPs shed the protein extra-domains of proteoglycans, and HPSE cleaves HS chains. (8) Soluble syndecans and HS fragments additionally activate inflammatory cells (9), which release cytokines. (10) Cytokines and growth factors activate endothelial cells to produce MMPs and HPSE, fueling the system.

One of the most important physiological roles of the glycocalyx is the recruitment of leukocytes to areas of infection with a multistep process: tethering, rolling, adhesion, and transmigration. Ordinarily, cell adhesion molecules on the endothelium, such as integrins and immunoglobulin glycoproteins, are hidden within the glycocalyx, but, once infection occurs, the glycocalyx is degraded by inflammatory mediators, which facilitate ligand–receptor interactions that promote the adhesion of leukocytes (Figure 1) [38].

## 3. Mechanisms of Endothelial Glycocalyx Damage

The mediators of glycocalyx degradation are copious, and some of them are interconnected, thus creating a vicious cycle: reactive oxygen/nitrogen species (ROS/RNS), matrix metalloproteinases (MMPs), hyaluronidase, and heparanase.

ROS and RNS are directly able to destroy GAGs; specifically, they induce modification in sugars and aid their hydrolytic cleavage [43,44]. Interestingly, hyaluronic acid (HA) is highly sensitive to chemical insults resulting in the generation of low-molecular-weight HA [45]. These species, in turn, activate a proinflammatory state, resulting in increased ROS/RNS production [46].

MMPs are a family of zinc-dependent endopeptidases that degrade extracellular matrix components (collagen, elastin, etc.). In the vasculature, MMPs are mainly expressed by inflammatory cells and, after specific stimuli, by endothelial and smooth muscle cells [47,48]. Their expression is regulated by ROS, cytokines, shear stress, and hypoxia [38]. In the glycocalyx, MMPs cleave the extracellular domains of syndecans. Syndecan-1 is shed by MMP-2, MMP-9, MT1-MMP, and ADAM-17 [39]. Syndecan-4 is shed by MMP-9 in a TNF-α-dependent manner [49]. MMPs are also able to cleave chondroitin sulfate [50].

Heparanase is an endo-β-D-glucuronidase, which cuts the heparan sulfate (HS) chains at the level of a limited number of specific intrachain sites, generating fragments of about 5–7 kDa [51]. Upregulation of heparanase expression in the vascular endothelium at the site of inflammation has been reported in multiple organ systems and promotes inflammatory responses [52]. Heparanase expression is upregulated in endothelial cells by several factors: ROS [53], inflammatory cytokines [54], high glucose [55], and advanced glycosylation products [56]. In the vascular district, heparanase is also released by inflammatory cells and platelet [57].

Heparanase can modulate glycocalyx damage in a manifold manner.

(1) By degrading HS, it modulates the interaction of endothelial cells with blood cells [58], regulates vascular permeability [59], and makes adhesion molecules on endothelium more accessible to circulating inflammatory cells [60].
(2) By releasing proinflammatory cytokines and chemokines bound to GAGs, it sustains inflammation, oxidative stress, and additional glycocalyx damage [61].
(3) Heparanase is also able to sustain inflammation by activating Toll-like receptors (TLRs) on macrophages via heparan sulfate fragments, leading to the activation of nuclear factor-κB (NF-κB), which results in the expression of additional inflammatory cytokines (TNF-α, IL-1β, and IL-8) [62]. The same cytokines can then sustain heparanase expression on endothelial cells [54] as well as the production of MMPs [63] and ROS.
(4) Heparanase also contributes to glycocalyx damage, thus increasing their procoagulant state by increasing tissue factor (TF) and modulating tissue factor pathway inhibitor (TFPI) [64].
(5) Lastly, the glypicans can also undergo shedding by phospholipase-D and notum (Figure 1) [65].

## 4. Glycocalyx Dysfunction Conditions

Pathological damage of the glycocalyx occurs in response to mechanical cellular stress, endotoxins, inflammatory mediators, atrial natriuretic peptide, ischemia/reperfusion injury, free oxygen radicals, and hyperglycemia, as well as the novel coronavirus disease 2019 (COVID-19) [49,66]. Degradation of the glycocalyx results in the shedding of some of its components in the blood flow (hyaluronic acid, heparan sulfate, syndecan-1 and 4, and glypicans), and often they correlate with the severity of particular diseases [50,67].

Glycocalyx degradation happens in infective (sepsis) and noninfective (trauma) inflammation [68], and in these settings, tissue necrosis factor-a (TNF-a) has a central role [69]. It exerts its role via heparanase activation and MMP9 upregulation [63,70]. Sepsis causes glycocalyx degradation but also delays its regeneration [71]. The breakdown of the endothelial glycocalyx stimulates macrophage recruitment and macrophage phenotype alterations [72,73] as well as leukocyte adhesion and focal vascular inflammation [74].

Collecting proof from in vitro and in vivo studies shows that hemorrhagic shock induces endothelial glycocalyx shedding and endothelial injury, accompanied by disturbed junctional integrity [75,76]. During ischemia/reperfusion (I/R) injury, it is damaged both during the hypoxic phase but also by the restoration of the blood supply (reperfusion). Endothelial cells after ischemia/reperfusion become swollen and detached from the basement membrane. Increased release of histamine and cathepsin, as well as oxygen free radicals and heparanase, may account for glycocalyx damage [77,78]. It has been proved that in patients undergoing major vascular surgery with global or regional ischemia, components of the glycocalyx, such as heparan sulfate and syndecan-1, are released into the plasma [79].

There is evidence that diabetes disturbs the vasculature globally, and alterations of the endothelial glycocalyx happen early in the onset of the disease [80]. It has been proved that acute hyperglycemia increases glycocalyx degradation and vascular barrier instability [81] confirmed also by in vitro studies [82].

The new coronavirus disease 2019 (COVID-19) caused by severe acute respiratory syndrome coronavirus 2 (SARS-CoV-2) in patients induces not only pulmonary disease, eventually culminating in acute respiratory distress syndrome (ARDS), but also produces multiple systemic effects, including acute kidney injury (AKI), acute cardiac injury, coagulopathy, thromboembolic complications, including stroke and pulmonary embolism, and circulatory shock [83]. Vascular endothelial damage has been identified as a common feature of high-risk patients prone to severe COVID-19, and several studies have shown that endothelial glycocalyx is seriously damaged during COVID-19, as proved by the increased shedding of syndecan-1 and hyaluronic acid and increased levels of heparanase [66,84].

## 5. Role of Glycocalyx Dysfunction in Fibrosis

Some recent findings indicate that mechanisms involved in endothelial glycocalyx dysfunction may participate in organ fibrosis, especially in renal fibrosis. Renal fibrosis is the final common outcome of practically all renal diseases causing chronic kidney disease (CKD). The main macroscopic characteristics of renal fibrosis are glomerulosclerosis, tubulointerstitial accumulation of extracellular matrix, inflammatory infiltration, tubular atrophy, capillary loss, and podocyte depletion. These events are caused by several biological events, including mesangial and fibroblast activation, monocyte/macrophage and T-cell infiltration, and cell apoptosis, which result in irreversible organ damage [85]. Activated myofibroblasts are the sources of extracellular matrix accumulation and they originate from several sources: interstitial renal fibroblasts, interstitial perivascular cells called pericytes, fibrocytes, tubular epithelial cells, and endothelial cells [86].

As described above, heparanase is an active player in glycocalyx remodeling, and the same agents that disrupt glycocalyx represent an element of damage and triggers renal fibrosis. Heparanase plasmatic levels are upregulated in chronic kidney disease patients [87], and several works have proved that heparanase is a central player in regulating the development and progression of renal fibrosis by modulating the epithelial-to-mesenchymal transition (EMT) of proximal tubular cells in diabetic conditions and after ischemia/reperfusion injury (I/R) [88,89,90,91]. We can therefore speculate that increased plasmatic heparanase levels can then sustain local fibrosis in damaged areas.

One of the actions of heparanase is the mobilization of cytokines and growth factors from the GAGs of the glycocalyx. Among them, TGF-β and FGF-2 are crucial elements that prompt fibrosis, and it has also been proved that heparanase sustains their induced EMT in the kidney [92,93]. Specifically, heparanase is necessary to maintain a rapid TGF-β effect and sustain its autocrine loop [92]. Heparanase is also necessary to activate FGF-2 signaling and maintain the autocrine loop by regulating MMP-dependent syndecan-1 shedding [93].

As described above, glycocalyx degradation is a crucial step for inflammatory cell recruitment, and heparanase is necessary for macrophage infiltration in diabetic nephropathy [91] and I/R injury [93]. It has been proved that heparanase polarizes macrophages toward an M1 proinflammatory and profibrotic phenotype in I/R injured kidney, and then it regulates the cross-talk between macrophages and tubular epithelial cells, which undergo EMT [94]. The recruitment of M1 macrophages then increases the production of profibrotic cytokines [94], which participate in the development of CKD and organ fibrosis. A recent study proved, in a mouse model, that heparanase inhibition prevented the development of chronic fibrosis in a model of I/R injury [88]. Overall, the present findings prove that heparanase, independently of the underlying nephropathy, regulates the development of fibrosis in chronic kidney disease modulating EMT and inflammation.

In the kidney, endothelial glycocalyx damage increased the production of vasoconstrictor agents [95]. Endothelin-1 (ET-1) is a potent endothelial-cell-derived vasoconstrictor. It is triggered by multiple stimuli such as ROS and inflammatory cytokines [96] and is released upon endothelial activation and activates two G-protein-coupled receptors, endothelin receptor type A (ETRA) and endothelin receptor type B (ETRB). Together, these receptors induce a variety of intracellular signaling cascades, resulting in vasoconstriction, proliferation, inflammation, extracellular matrix production, and fibrosis [97,98]. Recent findings have proved a renal interaction between heparanase and ET-1. Firstly, it was observed that endothelin-1 activates podocytes to release heparanase, thus causing damage to the glycocalyx, proteinuria, and renal failure [99]; secondly, it was observed that heparanase overexpression increases ET-1 levels after I/R renal injury [77], and heparanase inhibition reduces ET-1 expression [77,88] and its associated renal fibrosis [88]. This system, which leads to renal damage and fibrosis development, is also fueled by other agents, angiotensin II and aldosterone. Angiotensin II induces the production of ET-1 in podocytes and mesangial cells [95], increases heparanase expression, and, moreover, induces the production of aldosterone, which additionally increases heparanase expression [100].

An additional layer of evidence as to the involvement of heparanase in diabetic nephropathy is its levels observed in the urine of diabetic patients. Those (diabetics) who underwent kidney transplantation showed significantly lower urine heparanase levels compared to the ones who had not undergone a transplant [101]. Although it is known that glucose may regulate heparanase secretion [102], insulin seems to cooperate with glucose to promote heparanase secretion in HK-293 cells, though heparanase gene expression is inhibited by insulin in human aortic endothelial cells [103]. Moreover, heparanase per se has been shown to trigger the activation of the insulin receptor signaling pathway [104], leading to ERK activation [105], a well-known signaling pathway involved in EMT. Should this also occur in endothelial cells, a heparanase–insulin-dependent vicious cycle would further amplify/accelerate fibrotic processes. It should also be borne in mind that endothelial cells in insulin-resistant and/or diabetic subjects develop a selective insulin resistance whereby only the antiatherogenic IRS-PI3K-Akt arm responsible for NO production becomes resistant [106]. The other arm, the MAPK-ERK pathway, known to be proatherogenic, retains responsiveness to insulin. Thus, the selective loss of insulin action may further aid CVD.

Another capable linker between endothelial glycocalyx dysfunction and fibrosis is sirtuin-1 (SIRT1). Sirtuins (silent information regulator (SIRT)) are a group of NAD-dependent histone deacetylases that regulate chromatin silencing and transcriptional repression. Since they modulate several pathways, they are linked to cellular energy metabolism, mitochondrial biogenesis, stress response, apoptosis, inflammation, and also fibrosis [107]. The lack or reduction of SIRT1 impacts all the endothelial functions [106], but mice with endothelial deficiency of SIRT-1 develop tubulointerstitial fibrosis as well [108]. In this model, it has been described that SIRT-1 deficiency is associated with decreased MMP-14 levels [109]. It has also been documented that treatments with losartan, an angiotensin II receptor blocker, and hydrogen-rich water by increasing SIRT-1 reduce EMT and fibrosis in UUO mouse models [110,111].

**Figure 2:**
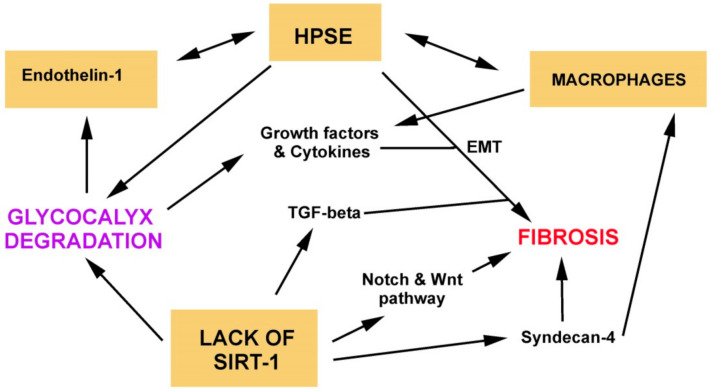
A series of noxa (ischemia/reperfusion (I/R), ROS, diabetes hyperinsulinemia, sepsis) increases heparanase production, which participates in glycocalyx degradation and the release of growth factors. Heparanase and these factors then sustain fibrosis via the activation of the EMT process. There is close interaction between macrophages and heparanase in the regulation of the fibrotic process. The production of heparanase is moreover sustained by a vicious loop with endothelin-1 released during glycocalyx degradation. In addition, the reduction of sirtuin-1 increases TGF-beta signaling fueling fibrosis via EMT. The lack of sirtuin-1 also induces fibrosis through the activation of the Notch and Wnt signaling pathways and the release of syndecan-4, which acts as a chemoattractant.

It has been proved that depletion of SIRT1 increases TGF-β1 activation, the acetylation of Smad3 [112], and that mice with endothelium-specific heterozygous TGFβ receptor II knockout (TGFβRIIendo+/−) are also protected against tubulointerstitial fibrosis via the inhibition of endothelial-to-mesenchymal transition [113]. Recently, it has been proved that a possible link between dysfunctional endothelial cells and the activation of fibroblasts, supporting fibrosis, could derive from specific factors secreted by endothelial cells with low (or lacking) SIRT-1 levels [108]. It has been found that endothelial cells lacking SIRT-1, when treated with TGF-β, release increased levels of Jagged1, Dickkopf-related protein 3 (DKK3), and syndecan-4 [108]. Jagged1 is a ligand of the Notch pathway, and DKK3 is a putative ligand of the Wnt pathway. Both pathways are implicated in the development of fibrosis [114]. The increased expression of syndecan-4 in endothelial cells lacking SIRT-1 is mediated via the NF-κB pathway. Normally, SIRT-1 deacetylates p65 and prevents its nuclear translocation [115], but when it is reduced, p65 increases the transcription of syndecan-4, which has NF-kB-response elements in its promoter regions [116]. The shedding and processing of heparan sulfate proteoglycan syndecan-4 are mediated by ADAM-17 and heparanase, and both are under NF-κB regulation. Lastly, syndecan-4 ectodomain accumulation in the interstitium acts as a macrophage chemoattractant, increasing fibroblast activation and inducing renal interstitial fibrosis (Figure 2) [117].

## 6. Therapeutic Strategies to Preserve Glycocalyx

Since glycocalyx degradation affects the normal functions of endothelium but also influences several pathological processes such as inflammation and fibrosis, there is the necessity of therapeutic intervention to preserve its integrity and to restore its structure. Below, we will briefly show the available and promising strategies to achieve this goal.

- Resuscitation fluids (fresh frozen plasma, plasma albumin, and hydroxyethyl starch) may influence glycocalyx shedding [118].
- It has been proved that anesthetic sevoflurane attenuates glycocalyx degradation in guinea pig hearts in a myocardial I/R injury model [78].
- Glucocorticoid: hydrocortisone reduced coronary resistance, vascular permeability, tissue edema, the release of lactate, uric acid, purines, and histamine, which were accompanied by severe degradation induced by TNF-α [119]. Dexamethasone suppressed the expression of MMPs and rescued the expression of ZO-1 and syndecan-1 in sepsis [120].
- Elevated levels of oxidative stress are present in the serum of CKD patients [121]; moreover, antioxidant elements such as ascorbic acid are reduced, limiting NO bioactivity [122]. Some strategies aimed at reducing oxidative stress have been tested. In a rat model of angiotensin-II-induced hypertension, the administration of green tea extract restored endothelium vasodilatation through ROS scavenging [123]. Additionally, the use of the antioxidant N-acetylcysteine reduces oxidative stress in a hyperglycemic state and, by doing so, reduces endothelial activation [124].
- Heparin and heparinoids may act toward several mechanisms. Firstly, heparins, by binding to endothelial cells, participate in the reconstitution of the glycocalyx and recover its negative charge [125]. It has also been reported that heparins increase heparan sulfate production and sustain its sulfation pattern [126]. Secondly, heparins are able to control multiple inflammatory effects. Heparins are able to protect cells from ROS, and they bind complement, growth factors and cytokines (i.e., interferon-γ and FGF-2), and P- and L-selectin (inhibiting leukocyte adhesion) [127]. Third, heparins protect endothelial cells from high-glucose damage by preventing the interaction of advanced glycosylation end products with their receptors [128], reducing membrane disruption and cell death [127]. Lastly, heparins are heparanase inhibitors, and thus they can modulate all the effects of this enzyme in direct glycocalyx degradation but also in inflammation and fibrosis [127]. Heparins are also able to bind and inhibit NF-κB [128] and thus regulate inflammatory cytokines but probably also the same heparanase and syndecan-4 expression involved in the development of fibrosis [88,116]. In this situation, a promising agent is sulodexide, a mixture of 80% fast-moving heparin and 20% of dermatan sulfate. Sulodexide has antithrombotic, profibrinolytic, anti-inflammatory, antioxidant, and anti-ischemic properties. In addition, its proposed mode of action is the inhibition of heparanase and also the modulation of MMP-9 production [127]. Animal models revealed multifaceted effects of sulodexide on endothelial functions [127,129], and, in clinical evaluation, sulodexide was able to partially restore endothelial glycocalyx and vascular permeability in patients with type 2 diabetes [130,131].
- Another element that could help to maintain glycocalyx integrity in diabetes is atrasentan and metformin. Atrasentan, antagonizing endothelin-1, reduces the glomerular expression of heparanase and its activator cathepsin-L [73]. The mechanism of action of metformin has not yet been clarified, but two weeks of metformin in drinking water is associated with an improvement in glycocalyx barrier properties in db/db mice [132].
- Since MMPs are central elements in glycocalyx degradation, some attempts at inhibition have been made, but more cell and animal experiments are necessary for a clinical translation. In vitro, sphingosine-1-phosphate (S1P) inhibits MMP-9 and -13 activity by activating the S1P1 receptor, which restores the endothelial glycocalyx through the activation of the PI3K pathway. S1P by inhibiting MMPs prevents the shedding of CS, HS, and the syndecan-1 ectodomain [133,134]. The use of pan-MMPs inhibitors, however, is not viable [135]. Some studies have shown that specific MMP-2 and -9 inhibition prevent the shedding of SDC-4 and HS in response to TNF-α preserving glycocalyx integrity [63,136].
- Another strategy to protect and reconstitute damaged glycocalyx is to supply endothelial cells with glycocalyx components. It has been proved, in an in vivo model, that glycocalyx damaged by hyaluronidase treatment can be partially recovered by acute infusion of hyaluronan and chondroitin sulfate [137]. It has also been proposed that the use of glycocalyx-mimetic biomaterials such as corline heparin conjugate, a structure resembling a proteoglycan, is able to protect the vasculature in thrombotic disorders and organ transplantation [138]. Additionally, elements designed to improve the compatibility between blood and polymeric biomaterials, such as the glycocalyx-mimetic dextran-modified poly(vinyl amine) surfactant, could represent useful tools to ameliorate glycocalyx structure [139].
- Possible strategies in future could implement NO production through the use of small molecules such as the protein kinase C inhibitor midostaurin, the pentacyclic triterpenoids ursolic acid and betulinic acid, the eNOS-enhancing compounds AVE9488 and AVE3085, and the polyphenolic phytoalexin transresveratrol [140].
- Giving the central role of SIRT-1 on endothelial glycocalyx preservation strategies aimed at restoration of its expression and activity are currently being tested [141]. The first generation of SIRT1 activators were plant polyphenols, such as butein, piceatannol, isoliquiritigenin, and mostly resveratrol [142,143]. Advances in sirtuin biochemistry, assays, and crystal structures allowed the development of more specific SIRT-1 modulators. Three small-molecule SIRT1 activators (SRT2104, SRT2379, and SRT3025) have been tested in clinical trials. All the compounds were well tolerated. In three studies, in elderly volunteers, healthy cigarette smokers, and type 2 diabetics, the compound SRT2104 had a beneficial effect on lipids, decreasing serum cholesterol, LDL levels, and triglycerides [139]. SRT2104 also reduced the LPS-induced release of inflammatory mediators and activation of coagulation [144]. Other studies have been carried out to test the anti-inflammatory effects of SRT2104 [141]. Starting from this evidence, the evaluation of these compounds’ effects on glycocalyx preservation and the regulation of fibrosis would be desirable to be made.
- It has been described that the patchy degradation of ESG is a result of the exocytosis of lysosome-related organelles. The control of excessive exocytosis could be achieved by sustaining NO production such as with NG-hydroxy-l-arginine, a nitric oxide intermediate [145].
- A new and promising strategy to obtain the restoration of glycocalyx is the recently described nanoliposomal carriers of preassembled glycocalyx [146]. These structures are able to bind to cells with degraded glycocalyx and restore NO production in endothelial cells, and they are able to induce a flow-induced vasodilatory response in perfused mesenteric arteries with a degraded glycocalyx [146].

## 7. Conclusions

In conclusion, since the endothelial glycocalyx has a crucial role in the development of organ fibrosis further research is needed to translate to the clinic new strategies to maintain and reconstitute glycocalyx integrity.
